# PI3K/Akt/mTOR Signaling Pathway as a Target for Colorectal Cancer Treatment

**DOI:** 10.3390/ijms25063178

**Published:** 2024-03-09

**Authors:** Premila D. Leiphrakpam, Chandrakanth Are

**Affiliations:** 1 Graduate Medical Education, College of Medicine, University of Nebraska Medical Center, Omaha, NE 68198, USA; 2 Division of Surgical Oncology, Department of Surgery, College of Medicine, University of Nebraska Medical Center, Omaha, NE 68198, USA

**Keywords:** colorectal cancer, PI3K, Akt, mTOR, cell survival, apoptosis, inhibitors, targeted therapy

## Abstract

In the last decade, pathway-specific targeted therapy has revolutionized colorectal cancer (CRC) treatment strategies. This type of therapy targets a tumor-vulnerable spot formed primarily due to an alteration in an oncogene and/or a tumor suppressor gene. However, tumor heterogeneity in CRC frequently results in treatment resistance, underscoring the need to understand the molecular mechanisms involved in CRC for the development of novel targeted therapies. The phosphatidylinositol 3-kinase/protein kinase B/mammalian target of the rapamycin (PI3K/Akt/mTOR) signaling pathway axis is a major pathway altered in CRC. The aberrant activation of this pathway is associated with CRC initiation, progression, and metastasis and is critical for the development of drug resistance in CRC. Several drugs target PI3K/Akt/mTOR in clinical trials, alone or in combination, for the treatment of CRC. This review aims to provide an overview of the role of the PI3K/Akt/mTOR signaling pathway axis in driving CRC, existing PI3K/Akt/mTOR-targeted agents against CRC, their limitations, and future trends.

## 1. Introduction

Colorectal cancer (CRC) is a significant cause of morbidity and mortality worldwide and the second leading cause of cancer mortality in the United States [1,2,3]. According to the Global Cancer Observatory (GLOBOCAN) 2020 estimates, there were approximately 1.9 million new cases and 916,000 deaths from CRC in 2020 globally [1]. In the United States, there will be an estimated 153,000 new cases and 53,000 deaths from CRC in 2023 [2,3]. Currently, the overall 5-year relative survival rate of CRC is estimated at 65% for all stages in the United States, and this survival rate decreases to 14% for metastatic CRC (mCRC) [2,3].

Surgery alone, or in combination with chemotherapy and radiotherapy, remains the primary treatment modality for localized CRC. However, less than 20% of mCRC cases achieve long-term recovery with surgery and chemotherapy [4]. For unresectable mCRC, systemic chemotherapy is the primary treatment modality. Within this modality, oxaliplatin-containing regimens, irinotecan-containing regimens, and fluorouracil-containing regimens are used for the first-line treatment of mCRC [4,5]. Although various improvements have been achieved in recent years, conventional therapeutic approaches are unable to eradicate all cancer cells due to the rapid evolution of drug resistance and cancer recurrence [5,6]. Tumor heterogeneity is one of the main mechanisms of drug resistance in CRC, mainly due to genetic and epigenetic mechanisms [6]. Hence, understanding the molecular complexity of CRC is required to develop other treatment modalities for CRC, especially for mCRC.

Over the past two decades, there has been a significant advancement in our understanding of the underlying molecular pathways involved in CRC pathogenesis. As a result, the treatment of CRC, particularly mCRC, has evolved significantly, reflected by the use of many chemotherapeutic combinations and the integration of novel targeted therapies into clinical practice [4,5,6]. This advancement has significantly improved the overall survival outcomes over time for mCRC patients participating in clinical trials [6,7].

Cetuximab, an anti-epidermal growth factor receptor (EGFR) monoclonal antibody, and bevacizumab, an anti-vascular endothelial growth factor (VEGF) monoclonal antibody, are among the first targeted agents for CRC that have been approved by the Food and Drug Administration (FDA) [4,5,6]. Since then, many other target-specific drugs for CRC treatment have been approved by the FDA [6]. However, the efficacy of these agents is often limited due to mutations leading to the activation of compensatory pathways at both upstream and downstream levels, making targeted therapy ineffective [5,6]. In particular, acquired therapeutic resistance to anti-EGFR monoclonal antibody treatment in patients with mCRC has been attributed to the various tumor-promoting mutations in rat sarcoma (*KRAS*), v-raf murine sarcoma viral oncogene homolog B1 (*BRAF*), and phosphatidylinositol 3-kinase catalytic subunit alpha *(PI3KCA*) genes [6]. Therefore, a better understanding of these signaling pathways and their related mechanisms of drug resistance in CRC will facilitate the development of novel therapeutic strategies to reduce resistance and enhance patient survival.

The phosphatidylinositol 3-kinase/protein kinase B/mammalian target of the rapamycin (PI3K/Akt/mTOR) signaling pathway axis is one such pathway that plays a major role in the regulation of cell growth critical in both normal and cancer cells [8,9]. The deregulation of the PI3K/Akt/mTOR pathway is frequently involved in CRC initiation, progression, and metastasis and plays a role in drug resistance [9]. This article aims to provide an overview of the role of the PI3K/Akt/mTOR signaling pathway axis in driving CRC, existing PI3K/Akt/mTOR-targeted agents against CRC, their limitations, and future trends.

## 2. Overview of PI3K/Akt/mTOR Signaling

PI3Ks belong to a family of plasma membrane-associated lipid kinases that can phosphorylate the 3′ hydroxyl group of phosphatidylinositol and phosphoinositide [9,10]. PI3Ks are divided into three classes, I, II, and III, based on their structures and functions [10]. Class I PI3Ks are the best characterized and generally coupled to extracellular stimuli. This class is further divided into class IA PI3Ks, which are activated by receptor tyrosine kinases (RTKs), G protein-coupled receptors (GPCRs), and certain oncoproteins such as the small G-protein rat sarcoma virus (RAS), and class IB PI3Ks, which are regulated exclusively by GPCRs [10,11,12]. Class 1A PI3Ks are divided into three subclasses (α, β, and δ), and class 1B is denoted as γ [12]. PI3Kα and PI3Kβ are ubiquitously expressed, and PI3Kδ and PI3Kγ are mainly found in leucocytes and blood vessels [11,12]. Class IA PI3Ks have been reported to be implicated in human cancer. Class IA PI3Ks are heterodimers containing a regulatory subunit (p85α, p55α, p50α, p85β, and p55δ) collectively referred to as p85 and a catalytic (CAT) subunit (p110α, p110β, and p110δ). The three catalytic subunits are encoded by the *PIK3CA*, *PIK3CB*, and *PIK3CD* genes. Each of these three catalytic isoforms forms a dimer with a regulatory subunit and modulates the activation and subcellular localization of the complex [12].

**Figure 1:**
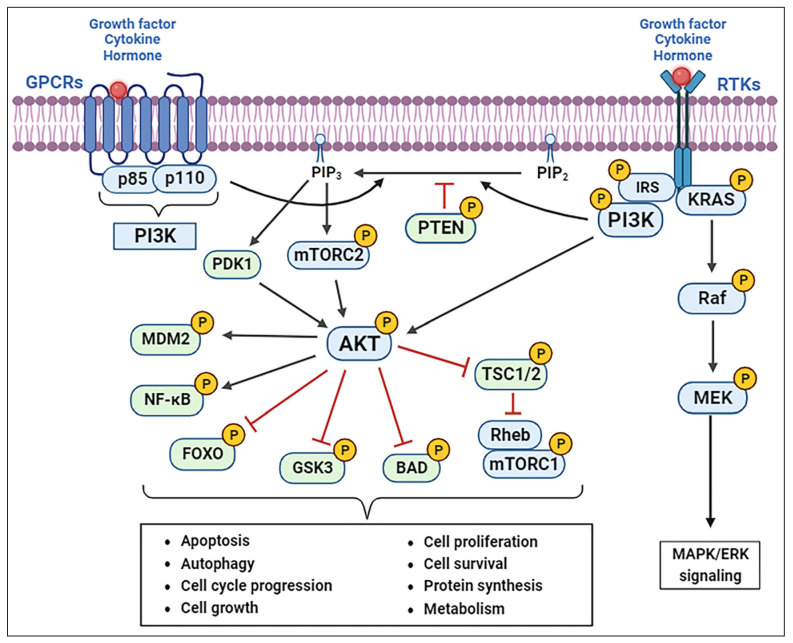
Schematic of the PI3K/Akt/mTOR signaling pathway. Akt, protein kinase B; BAD, Bcl2-associated agonist of cell death; CRC, colorectal cancer; ERK, extracellular signal-regulated kinase; FOXO, forkhead box O; GSK3, glycogen synthase kinase-3, KRAS, kirsten rat sarcoma virus; MAPK, mitogen-activated protein kinase; MEK, mitogen-activated protein kinase kinase; MDM2, mouse double minute 2 homolog; mTOR, mammalian target of rapamycin; mTORC1, mammalian target of rapamycin complex 1; mTORC2, mammalian target of rapamycin complex 2; NF-κB, nuclear factor-kappa B; PDK1, phosphoinositide-dependent kinase 1; PI3K, phosphatidylinositol 3-kinase; PTEN, phosphatase and tensin homolog; RAF, rapidly accelerated fibrosarcoma. TSC1/2, tuberous sclerosis proteins 1/2. Created with BioRender.com (accessed on 28 December 2023).

The PI3K pathway is activated by the RTKs in a tightly controlled, multistep process, as shown in Figure 1. Ligand binding activates RTKs such as the epidermal growth factor receptor (EGFR), the human epidermal growth factor receptor (HER2), the insulin receptor (IR), the insulin-like growth factor 1 receptor (IGF1R), the fibroblast growth factor receptor (FGFR), etc. [9]. These transmembrane receptors do not bind or activate PI3K directly; they phosphorylate adaptor proteins such as insulin receptor substrates 1 and 2 (IRS1, IRS2), growth factor receptor-bound protein 2 (Grb2)-associated binder ½ (GAB1/2), and rat sarcoma virus (RAS) family members [13]. These adaptor proteins then bind to the amino-terminal domain of the p85 regulatory subunit of PI3K, relieving the inhibition of p110 and recruiting the p85-p110 heterodimer to the membrane-bound phosphatidylinositol-(4,5)-bisphosphate (PIP_2_) [10,11]. This triggers the activation of PI3K and the conversion of PIP_2_ to phosphatidylinositol-(-3,4,5)-triphosphate (PIP_3_). PI3K can also be stimulated by activated RAS by binding directly to the Ras-binding domain in p110α [9,10,11]. PIP_3_ is a second messenger that recruits kinases with a pleckstrin homology domain (PH-domain) to the membrane and is inactivated through dephosphorylation by the phosphatase and tensin homolog (PTEN) [9,10,11]. 

PI3K-generated PIP_3_ binds to the PH domain of both Akt and phosphoinositide-dependent kinase 1 (PDK1) and induces their translocation to the plasma membrane, where PDK1 phosphorylates the Akt kinase at the Threonine 308 residue in the “activation loop,” leading to its partial activation [14]. This Akt modification then activates mTORC1 by directly phosphorylating and inactivating the proline-rich Akt substrate of 40 kDA (PRAS40) and tuberous sclerosis protein 2 (TSC2) [15]. The substrates of mTORC1, ribosomal protein S6 kinase polypeptide 1 (S6K1), and eukaryotic translation initiation factor 4E binding protein 1 (4RBP1), in turn, phosphorylate the ribosomal protein S6 (S6/RPS6), promoting protein synthesis and cellular proliferation [16].

Full Akt activity is achieved through phosphorylation at the Serine 473 residue in the C-terminal hydrophobic motif by mTORC2 [17]. This leads to downstream substrate-specific phosphorylation in both the cytoplasm and nucleus, including the Bcl2-associated agonist of cell death (BAD), forkhead box O (FOXO), glycogen synthase kinase 3 (GSK3), the mouse double minute 2 homolog (MDM2), and nuclear factor-kappa B (NF-κB) [16,17,18]. In normal cells, the fully activated PI3K/Akt/mTOR pathway influences many intracellular pathways that regulate cell survival, proliferation, growth, motility, metabolism, protein synthesis, transcription, apoptosis, and angiogenesis [16,17,18], as shown in Figure 1.

## 3. PI3K/Akt/mTOR Pathway Alteration in CRC

The PI3K/Akt/mTOR pathway axis is one of the most frequently altered pathways in human cancers [9]. Aberrant PI3K signaling is critical in driving tumor initiation and progression through the dysregulation of several cellular processes, such as proliferation, growth, apoptosis, survival, and cytoskeletal rearrangement, in many cancers, including CRC [9,10,11]. This aberrant activation of the PI3K/Akt/mTOR pathway involves processes such as activation by RTKs and the somatic mutation or amplification of genes encoding key components of the signaling pathway such as *PI3KCA*, *IRS1*, *PI3KR1*, *PDK1*, *AKT1*, *AKT2*, *PAK4*, and *mTOR* or a loss of *PTEN* and *TSC1/2* [19,20,21,22,23,24,25,26]. 

Somatic missense mutations in the *PIK3CA* gene are prevalent in approximately one-third of CRC cases at the adenoma–carcinoma transition and can lead to the direct activation of the PI3K/Akt/mTOR pathway [19,27]. Gain-of-function mutations in the exon 9 (E542K, E545K) helical domain and exon 20 (H1047R) kinase domain of the *PIK3CA* gene account for 80% of all *PIK3CA* gene mutations, resulting in the activation of this PI3K and its downstream signaling pathway [11,19,20,21]. The amplification of the PI3KCA gene through increased protein expression levels and activity is another mechanism of PI3K activation [19,20,21]. The *PIK3CA* gene mutation has been shown to increase CRC cell survival and proliferation, leading to chemotherapy resistance and a poor prognosis [28]. *PTEN*, a negative regulator of PI3K signaling, activates PI3K signaling via loss-of-function mutations in exons 7, 8, and 9, resulting in truncation and loss of phosphatase activity, deletion through homozygous or hemizygous allelic losses at chromosome 10q23, and epigenetic silencing via promoter hypermethylation in MSI-high tumors [20,21,22]. PI3Ks can also be activated by the genetic amplification of *IRS2*, an upstream activator of PI3K signaling, and a gain-of-function mutation of *PIK3R1* [20,21,24]. Gain-of-function mutations in the downstream mediators of PI3K signaling, such as *AKT1* in the PH domain (E17K), *PDK1, AKT2*, and *PAK4* in the kinase domains, and *mTOR*; the co-amplification of *AKT2* and *PAK4* on chromosome 19q13.2; and loss-of-function mutations in *TSC1/2* can also activate PI3K signaling [20,23,25,26]. Table 1 summarizes the key genetic alterations reported in the PI3K signaling pathway in CRC.

Akt is the dominant effector of several downstream signaling proteins activated by PI3K signaling and has been extensively studied. Akt1 and Akt2 isoforms have been reported to be mutated in CRC [20]. Akt regulates mTOR, a downstream target that promotes protein translation, growth, metabolism, and angiogenesis in CRC [29]. Furthermore, Akt promotes cell survival in CRC through the regulation of various downstream pro-survival targets, such as NF-κB, XIAP, and survivin, and inhibiting pro-apoptotic targets such as Bad, procaspase-9, FOXO, GSK3 β, and p53 [30,31]. 

Mutations in the PI3K/Akt/mTOR signaling axis are also associated with advanced cancer or metastasis, indicating the potential role of these mutations in cancer cell invasion and migration to distant sites [32]. Metastatic CRC (mCRC) lesions have been reported to have a higher frequency of exon 9 and 20 PIK3CA mutations compared to primary lesions [33]. However, aberrant PI3K expression in metastatic tumors is not always associated with PIK3CA mutations, suggesting the involvement of other mechanisms. We have studied the expression status of upstream and downstream markers of the PI3K/Akt signaling pathway in CRC, such as phosphatase of regenerating liver 3 (PRL3), an upstream marker of the PI3K/Akt pathway in CRC [34]. PRL3 activates PI3K/Akt signaling via PTEN inhibition and Akt activation [35]. The overexpression of PRL3 promotes cancer cell survival, migration, invasion, and metastasis through the activation of Akt [36]. Consistent with these findings, we demonstrated a significant positive correlation of PRL3 expression with activated Akt in mCRC [34]. We have also shown that both Ezrin and NHERF1 are downstream of the insulin growth factor 1 receptor (IGR-1R)/PI3K/Akt signaling axis and regulate CRC cell survival in vitro through the modulation of cell survival markers, XIAP, and survivin [37,38,39]. PRL3, ezrin, and NHERF1 are also highly expressed in patients with mCRC with minimal expression in normal or premalignant adenoma [34,37,38] and might be potential targets for anti-PI3K/Akt-mediated targeted therapy. 

## 4. Recent Advances in PI3K/Akt/mTOR Inhibitor Research for CRC Treatment

**Figure 2:**
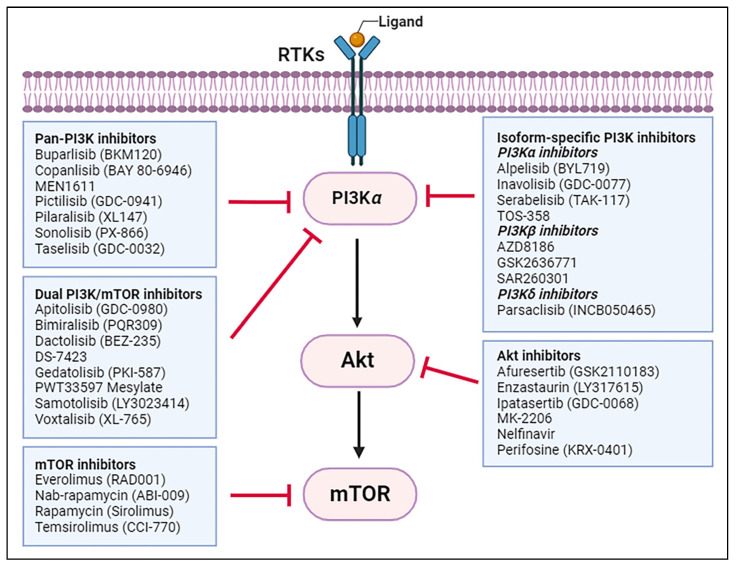
PI3K/Akt/mTOR targeted therapy for CRC. Created with BioRender.com (accessed on 1 March 2024).

The PI3K/Akt/mTOR pathway axis is critical for cell survival, proliferation, and growth, and it is one of the most frequently altered pathways in human cancer, making it a promising target for cancer therapy [9,10,11,12]. Cancer cells with a loss of PTEN function or hyperactivate PI3Ks are potentially sensitive to PI3K, Akt, and mTOR inhibitors [11]. Several drugs targeting the PI3K/Akt/mTOR pathway have been investigated for the treatment of CRC in different stages of clinical development, alone or in combination. They can be broadly classified into five different classes: Pan-PI3K inhibitors, isoform-specific PI3-K inhibitors, dual PI3K–mTOR inhibitors, Akt inhibitors, and mTOR inhibitors. Figure 2 depicts a schematic diagram of PI3K/Akt/mTOR-targeted drugs that have completed or are undergoing clinical trials for CRC treatment. 

### 4.1. Pan-PI3K Inhibitors

The pan-PI3K inhibitors are competitive ATP inhibitors that target all isoforms (α, β, δ, and γ) of class IA PI3Ks in cancer [10,11]. Wortmannin and LY294002 are the two prototypes of pan-PI3K inhibitors widely investigated in pre-clinical models but never fully developed as anticancer drugs for clinical use due to their sub-optimal pharmacokinetic properties [40]. Selected clinical trials of pan-PI3K inhibitors as monotherapy or in combination with other targeted agents to evaluate the benefit of blocking PI3Ks for CRC treatment are shown in Table 2.

Buparlisib (BKM120) is an oral pan-class I reversible inhibitor of PI3Ks that targets all four isoforms of Class I PI3Ks (α, β, γ, and δ) and has no inhibitory activity against the class III PI3Ks or mTOR [64]. In preclinical studies, buparlisib demonstrated a potent antiproliferative effect in human cancer cell lines bearing *PI3KCA* mutations [64] and significant antitumor activity at a tolerated dose in human tumor xenograft models with or without *PI3KCA/PTEN* mutations [41]. Several phase I/II trials of buparlisib alone or in combination with CRC have been investigated [41,42,43,44,45,46,47,48,49,50] (Table 2). A first-in-human phase I dose escalation study (NCT01068483) of buparlisib in patients with advanced solid tumors (including 31 patients with CRC) reported a favorable pharmacokinetic profile, consistent pharmacodynamic effects, and preliminary antitumor activity [41,42]. Another phase I study (NCT01591421) of buparlisib in combination with panitumumab showed tolerability, but a lack of efficacy in patients with KRAS WT advanced CRC [49]. 

Copanlisib (BAY 80–6946) is an intravenous, potent, and highly selective pan-class I PI3K inhibitor with predominant activity against the p110α and p110δ isoforms [65]. The first-in-human phase I study of copanlisib monotherapy (NCT00962611) in patients with advanced solid tumors and non-Hodgkin lymphomas showed promising anti-tumor activity, especially in patients with non-Hodgkin lymphoma [51]. Another phase I study (NCT01404390) of copanlisib in Japanese patients with advanced or refractory solid tumors demonstrated near-dose-proportional pharmacokinetics and preliminary disease control, warranting further investigation [52]. A phase I/II trial (NCT03711058) is currently underway to evaluate the effect of copanlisib and an anti-PD-1 antibody, nivolumab, in combination in relapsed or refractory solid tumors with expansions in mismatch-repair-proficient (MSS) CRC [54]. Another phase I/II trial (NCT04317105) is also currently underway to study the side effects and the optimal dose of copanlisib when given together with nivolumab and ipilimumab and to see how well they work in treating patients with advanced solid cancers with alterations in the *PIK3CA* and *PTEN* genes.

Other pan-PI3K inhibitors such as MEN1611 [55], pictilisib (GDC-0941) [56,57,58], pilaralisib (XL147) [59,60,61], sonolisib (PX-866) [62], and taselisib (GDC-0032) [63] have also been investigated in several phase 1 and II trials for CRC treatment (Table 2). The use of pan-PI34K inhibitors was restricted because of the adverse pharmacological events caused by off-target effects and the on-target consequences of blocking all class I PI3K isoforms, independent of their role in carcinogenesis [66]. Therefore, isoform-specific PI3K inhibitors are being investigated to reduce toxicity profiles and obtain complete target inhibition.

### 4.2. Isoform-Specific PI3K Inhibitors

Isoform-specific PI3K inhibitors selectively inhibit p110 or catalytic subunits [10]. A possible advantage of isoform-specific inhibitors is improved tolerance, resulting in more complete target inhibition with fewer adverse effects [10]. Selected clinical trials of isoform-specific PI3K inhibitors as monotherapy or in combination with other targeted agents to evaluate the benefit of blocking PI3Ks in CRC are shown in Table 3.

Alpelisib (BYL719) is an oral, highly selective small-molecule PI3Kα isoform inhibitor that selectively inhibits p110α [75]. Preclinical studies have reported the favorable antitumor activity of alpelisib in xenograft tumor models with an altered *PIK3CA* gene (mutation or amplification) [75]. The FDA approved alpelisib for the treatment of men and postmenopausal women with hormone receptor (HR)-positive and HER2-negative, *PIK3CA*-mutated, advanced breast cancer who have received endocrine therapy previously (SOLAR-1 phase II trial; NCT02437318) [76,77,78]. The trial reported a 7.9-month improvement in the median overall survival of breast cancer patients treated with alpelisib plus fulvestrant compared to patients treated with fulvestrant alone (39.3 months versus 31.4 months) [78]. The trial also reported a prolongation of overall survival in cancer patients with lung and/or liver metastases of up to 14 months and highlighted the importance of the potential application of alpelisib in the treatment of other PI3KCA-mutated metastatic solid tumors, including CRC [77]. A phase I dose-escalation study (NCT01219699) of alpelisib in patients with PIK3CA-altered advanced solid tumors, including CRC, demonstrated a tolerable safety profile and encouraged preliminary activity [60]. Another phase I/II trial (NCT04753203) with alpelisib and capecitabine is currently underway for PI3K-mutated mCRC. Furthermore, clinical studies also reported that the combined treatment of encorafenib plus cetuximab and alpelisib is tolerable and provides promising clinical activity in the difficult-to-treat patient population with BRAF-mutant mCRC (NCT01719380) [68,79].

Serabelisib (TAK-117) is another potent oral PI3Kα isoform inhibitor. The first-in-human phase I trial (NCT01449370) dose-escalation study of serabelisb in patients with advanced solid tumors, including CRC, reported an acceptable and manageable safety profile [70]. Phase I/II trials (NCT04073680, NCT05300048) of combination therapies of serabelisib with other agents are currently underway for treating advanced solid tumors with *PIK3CA* or *KRAS* mutations, including CRC. 

ADZ8186 is a potent and selective PI3Kβ/δ inhibitor, with the first-in-human phase I study (NCT01884285) characterizing its favorable safety and tolerability in patients with advanced solid tumors, including CRC, with common adverse events of diarrhea, nausea, and vomiting [71]. Another phase I study (NCT03218826) on the combination of AZD8186 and docetaxel is currently underway to evaluate the efficacy and safety of this combination in solid tumors. 

Another PI3Kβ inhibitor, GSK2636771, is currently in development for solid tumors, and the first-in-human study phase I/II study (NCT01458067) of GSK2636771 showed anti-tumor activity in patients with *PTEN*-deficient tumors [72]. The trial also reported that the combination of GSK2636771 and pembrolizumab showed an acceptable safety and tolerability profile in patients with *PTEN*-deficient advanced solid tumors [73]. Another phase II trial (the MATCH Screening Trial; NCT02465060) of GSK2636771 is currently being conducted for patients with advanced refractory solid tumors with *PTEN* loss.

### 4.3. Dual PI3K/mTOR Inhibitors

The catalytic domains of the p110 subunits and mTOR are structurally similar because they all belong to the phosphatidylinositol kinase-related family of kinases [80]. Many chemical inhibitors under development inhibit both mTOR and the p110 catalytic subunits and are termed dual PI3K/mTOR inhibitors. Compared with other PI3K pathway inhibitors, dual PI3K/mTOR inhibitors can inhibit all PI3K catalytic isoforms, mTORC1, and mTORC2 [80] and should block this pathway completely and overcome the feedback inhibition observed with mTORC1 inhibitors (i.e., rapamycin analogs). However, it remains unknown if dual PI3K/mTOR inhibitors will be tolerable at doses that effectively inhibit all p110 isoforms and mTOR. Selected clinical trials of dual PI3K/mTOR inhibitors as monotherapy or in combination with other targeted agents in CRC are highlighted in Table 4.

Dactolisib (BEZ-235) is a potent dual PI3K/mTOR inhibitor that inhibits PI3K and mTOR kinase activity by binding to the ATP-binding pockets of these enzymes [96]. Dactolisib inhibits mTORC1 and mTORC2 kinases at low doses, whereas at high doses, it inhibits mTOR and all class I PI3Ks [96]. Both in vitro and in vivo data have shown that dactolisib is a potent dual PI3K/mTOR modulator with favorable pharmaceutical properties and antitumor activity [96]. Dactolisib has also been investigated in various phase I and II dose escalation studies (NCT00620594, NCT01195376, NCT01285466, NCT01337765, NCT01343498, NCT01508104), both as monotherapy and combination therapy in advanced solid tumors [83,84,85,86,87]. Although robust antitumor activity was demonstrated in preclinical studies, dactolisib was found to have a limited level of clinical activity and highly variable pharmacokinetic characteristics in these studies, and further development of the drug in oncology indications has been discontinued [25,83].

Gedatolisib (PKI-587) is an intravenous, potent, and highly selective dual PI3K/mTOR inhibitor. A first-in-human phase I study (NCT00940498) of gedatolisib in advanced solid tumors demonstrated a manageable safety profile and antitumor activity [89]. However, a phase I (NCT01347866) combination study of gedatolisib and irinotecan showed limited activity in patients with advanced CRC [90].

Samotolisib (LY3023414) and voxtalisib (XL-765) are selective dual PI3K/mTOR inhibitors that showed tolerable safety profiles and single-agent activity in patients with advanced cancers in phase I trials (NCT01655225, NCT00485719) [91,93]. Despite these results, combination studies of samotolisib (NCT02784795) and voxtalisib (NCT00777699, NCT01390818) showed poor tolerability and/or limited anti-tumor activity in patients with advanced or metastatic solid tumors [92,94,95].

### 4.4. Akt Inhibitors

Akt inhibitors can be broadly categorized as ATP-competitive inhibitors, phosphatidylinositol analogs, and allosteric inhibitors [66]. Cancers with AKT1 mutations and AKT1 and AKT2 amplifications are expected to be among the most sensitive to Akt inhibitors [10]. However, this class of inhibitors will not block the non-Akt effectors of PI3K signaling and could reciprocally increase the PI3K-dependent activation of those effectors via a loss of negative feedback [10]. Moreover, low activity of Akt inhibitors has been reported in PI3KCA-mutated cancers, mainly due to an Akt-independent mechanism [97]. Several Akt inhibitors are in different clinical stages for various cancers, including CRC. Table 5 highlights selected clinical trials of Akt inhibitors for CRC treatment.

MK-2206, an oral allosteric inhibitor of all Akt isoforms, prevents the downstream activation of Akt substrates by inhibiting the translocation of Akt to the membrane [25]. MK-2206 showed antitumor activity in preclinical studies for CRC [31], and a first-in-human phase 1 trial (NCT00670488) of MK-2206 reported a favorable toxicity profile with evidence of Akt signaling blockade [101]. However, phase II trials (NCT01333475, NCT01802320) of MK-2206 monotherapy or in combination with MEK inhibitors in mCRC did not achieve the optimal level of target inhibition [102,103]. 

Perfosine is a novel orally bioavailable alkylphospholipid compound that inhibits Akt phosphorylation [121]. A phase II trial (NCT00398879) of perifosine with capecitabine in 38 patients with previously treated mCRC showed promising clinical activity compared with a placebo plus capecitabine [104]. Despite this promising data, the phase III XPECT trial (NCT01097018) showed no benefit in overall survival with a combination of perifosine and capecitabine in refractory mCRC [105].

### 4.5. mTOR Inhibitors

mTOR plays a critical role in PI3K/Akt/mTOR signaling axis-mediated tumor progression and is one of the main molecular targets of cancer inhibitors targeting this pathway axis. mTOR inhibitors can be classified as rapamycin and its analogs (rapalogs), which block the activity of mTORC1, ATP-competitive mTOR inhibitors that inhibit both mTORC1 and mTORC2 and dual PI3K/mTOR inhibitors, as discussed in Section 4.3 of the review article [80,122]. Rapamycin and rapalogs interact with the intracellular receptor of FK506 binding protein 12 (FKBP12) in mammalian cells to form a complex with a high affinity for mTOR in mTORC1 but not mTORC2 [80,122]. This could result in losing the mTORC2-mediated feedback inhibition of Akt, promoting cell survival and chemoresistance [122]. However, ATP-competitive mTOR inhibitors target mTOR’s kinase domain and can inhibit rapamycin-sensitive mTORC1 and rapamycin-insensitive mTORC2, resulting in a robust anti-cancer effect [122,123,124]. These dual kinase inhibitors are more potent than rapalogs, as the inhibition of mTORC2 blocks Akt activation and might mitigate the negative feedback activation of PI3Ks that often accompanies mTORC1 inhibition [10]. Therefore, ATP-competitive mTOR inhibitors might be more effective than rapamycin and its analogs. Table 5 highlights selected phase I and II trials of mTOR inhibitors evaluated for treating CRC.

Rapamycin showed broad anticancer activity; however, its clinical development was hindered due to its unfavorable pharmacokinetic properties [27]. Rapalogs such as everolimus and temsirolimus have been approved for treating breast cancer, neuroendocrine tumors, and renal cell carcinoma [27]. Several phase I/II trials have also investigated the utility of rapamycin and raplogs in the treatment of mCRC [106,107,108,109,110,111,112,113,114,115,116,117,118,119,120]. Currently, no mTOR inhibitors are available for CRC treatment; however, several dual mTORC1/2 kinase inhibitors in pre-clinical studies have shown potential [125,126,127,128] and could effectively alleviate the feedback activation of Akt in CRC treatment. A recent preclinical study reported that sapanisertib (TAK-228), a dual TORC1/2 inhibitor, can overcome resistance to everolimus and induce a treatment response in *PIK3CA* mutant CRC in spheroids, isogenic human cell lines, and in a transgenic mouse model [127]. Further investigation in clinical trials is required to determine the full potential of dual TORC1/2 inhibitors in CRC treatment. 

## 5. Discussion

The aberrant PI3K/Akt/mTOR pathway axis is associated with tumorigenesis, tumor progression, and drug resistance [129]. However, the complex nature of the PI3K/Akt/mTOR pathway due to its multiple levels of feedback and crosstalk with other pathways has challenged the full effect of PI3K inhibitors in the treatment of cancer patients [130]. The dependence of tumor cells on multiple oncogenic pathways and poor tolerability could contribute to the failure of monotherapy with pan-PI3K and mTOR inhibitors in solid cancers, including CRC [130]. Studies have shown that PI3K inhibitors are likely more effective in cancers with mutations in the PI3K pathway, including CRC [131,132]. It is, therefore, crucial to develop inhibitors of the PI3K/AKT/mTOR pathway with rational targets in mind, such as *PTEN* loss and *PIK3CA*-activating mutations, in combination with downstream molecular marker evaluations, which is more likely to yield success than current approaches in CRC treatment. Optimization strategies for patient selection, such as identifying patient cohorts harboring *PI3KCA* mutations, will also benefit treatments.

The PI3K/Akt/mTOR pathway bifurcates at PI3K/Akt and integrates with signaling molecules from other pathways [16]. Therefore, targeting Akt might have a global effect as it is less susceptible to potential feedback loops compared to targeting molecules further downstream. However, a patient with an activating mutation in a downstream component of the pathway might not respond to a drug targeting an upstream component. Hence, identifying novel molecules at different levels of the PI3K signaling pathway is critical for understanding the precise mechanism of this pathway and its inhibition and for developing therapeutic strategies to enhance the efficacy of PI3K inhibitors or to replace PI3K inhibitors. 

The crosstalk between the PI3K/Akt/mTOR and RAS/RAF/MAPK pathways in CRC is well documented and is one of the main resistance mechanisms in CRC treatment [6]. The EGFR pathway, in particular, signals through these two pathways, and the interaction between these two pathways is one of the mechanisms involved in anti-EGFR therapy resistance in CRC [133]. Recent clinical trials have reported that metastatic CRC with tumor-promoting mutations in *KRAS*, *PI3KCA*, and *BRAF* is resistant to anti-EGFR therapy. In addition, activating mutations in *BRAF* and *PIK3CA* may be partially responsible for the failure of anti-EGFR therapy in KRAS-WT CRC patients [134]. It has been reported that the inhibition of the Wnt/β-catenin pathway is associated with the upregulation of the PI3K/AKT/mTORC1 pathway in CRC, and blocking the PI3K/AKT/mTORC1 pathway results in Wnt/β-catenin signaling hyperactivation as a compensatory mechanism [135]. Studies have also shown the impact of the PI3K pathway on immune cells in many cancers, including CRC [136,137]. CRC tumor cells with PI3KCA mutations or PTEN loss have been shown to be associated with increased PDL-1 expression, a key immune checkpoint molecule, thereby conferring resistance to anti-PD1 therapy [137]. This resistance has been shown to be related to the immune evasion process in tumor cells despite increased engagement, and the coadministration of PDL-1 and PI3K inhibitors has been shown to reverse this resistance [137]. Other pathways, such as the TGFβ [138] and NOTCH/MYC pathways, also modulate PI3K/AKT/mTOR-targeted therapy [139]. Hence, modulation of PI3K signaling inhibition is required to improve the efficacy of therapeutics that target various signaling pathways, including RTK signaling, in CRC treatment [140].

Other challenges of PI3K/Akt/mTOR-targeted therapy include drug-related adverse events and/or direct-drug toxicity, such as hyperglycemia, diarrhea, nausea, vomiting, cutaneous reactions, hypertension, hepatotoxic effects, and neuropsychiatric problems [41,141]. PI3K signaling plays an important role in insulin signaling and glucose homeostasis, and the inhibition of this pathway leads to insulin resistance (manifested as hyperglycemia) and is due to the feedback activation of insulin signaling [142]. This feedback mechanism can be prevented with dietary or pharmacological approaches [142], and trials are underway for advanced solid tumors with *PIK3CA* mutations (NCT04073680, NCT05300048). In addition, isoform-selective PI3K inhibitors have been reported to be well tolerated compared to pan-PI3K inhibitors and mTOR inhibitors [130].

Thus, the focus should be on optimizing strategies of patient-targeted therapy and combination therapies in CRC to increase efficacy and minimize resistance.

## 6. Conclusions

Significant advancements have been achieved in treating CRC; however, treatment resistance is a considerable setback, mainly in mCRC. The aberrant PI3K/Akt/mTOR signaling pathway is a major resistance mechanism to CRC therapy. Therefore, understanding the precise mechanisms of the PI3K/Akt/mTOR signaling pathway axis and elucidating the underlying PI3K-mediated resistance mechanism can provide a rationale for combination and patient-targeted therapies in CRC. 

## Figures and Tables

**Table 1:** Genetic alterations in the PI3K signaling pathway in CRC.

Gene	Alteration	Expected Outcome	References
*PIK3CA*	Mutation	Gain of function	[19,20,21]
	Amplification		
*PTEN*	Mutation	Loss of function	[20,21,22]
	Deletion		
	Epigenetic silencing		
*IRS2*	Amplification	Gain of function	[20]
*PIK3R1*	Mutation	Gain of function	[24]
*PDK1*	Mutation	Gain of function	[20]
*AKT1*	Mutation	Gain of function	[23]
*AKT2*	Mutation	Gain of function	[20]
	Co-amplification		
*PAK4*	Mutation	Gain of function	[20]
	Co-amplification		
*mTOR*	Mutation	Gain of function	[25]
*TSC1/2*	Mutation	Loss of function	[25,26]

Abbreviations: *AKT1*, AKT serine/threonine kinase 1; *AKT2*, AKT serine/threonine kinase 2; *IRS2*, insulin receptor substrate 2; *mTOR*, mammalian target of rapamycin; *PAK4*, p21-activated kinase 4; *PDK1*, phosphoinositide-dependent protein kinase-1; *PI3KCA*, phosphatidylinositol 3-kinase catalytic subunit; *PIK3R1*, phosphatidylinositol 3-kinase regulatory subunit 1; *PTEN*, phosphatase and tensin homolog; *TSC1/2*, tuberous sclerosis protein 1/2.

**Table 2:** Selected phase I and II trials of pan-PI3K inhibitors for CRC treatment.

Drug	Condition	Phase	Status	NCT Number, Reference
Buparlisib (BKM120)	Advanced solid tumors	I	Completed	NCT01068483 [41,42]
	Advanced solid tumors	I	Completed	NCT01155453 [43]
	Advanced solid tumors	I	Completed	NCT01285466 [44]
	Advanced CRC	I	Completed	NCT01304602 [45]
	Advanced solid tumors	I	Completed	NCT01363232 [46]
	*PIK3CA* mutated cancers	I	Withdrawn	NCT01501604
	Advanced solid tumors	I	Completed	NCT01571024 [47]
	Advanced solid tumors	I	Completed	NCT01576666 [48]
	RAS-WT mCRC	I/II	Completed	NCT01591421 [49]
	Advanced solid tumors	I	Completed	NCT01626209 [50]
Copanlisib (BAY 80-6946)	Advanced tumors	I	Completed	NCT00962611 [51]
	Advanced or refractory solid tumors	I	Completed	NCT01404390 [52]
	Advanced or refractory solid tumors	II	Active, not recruiting	NCT02465060 [53]
	Relapsed or refractory solid tumors with expansions in MSS CRC	I/II	Active, not recruiting	NCT03711058 [54]
	*PIK3CA* and *PTEN* mutated advanced solid tumors	I/II	Active, not recruiting	NCT04317105
MEN1611	mCRC	I/II	Active, not recruiting	NCT04495621 [55]
Pictilisib (GDC-0941)	Locally advanced or metastatic solid tumors	I	Completed	NCT00876122 [56]
	Advanced solid tumors	I	Completed	NCT00975182 [57]
	Locally advanced or metastatic solid tumors	I	Terminated	NCT00996892 [58]
Pilaralisib (XL147)	Advanced solid tumors	I	Completed	NCT00486135 [59,60]
	Solid tumors	I	Completed	NCT00756847 [61]
	Locally advanced or metastatic solid tumors	I	Completed	NCT01357330
Sonolisib (PX-866)	mCRC	I/II	Completed	NCT01252628 [62]
Taselisib (GDC-0032)	Advanced refractory solid tumors	II	Active, not recruiting	NCT02465060 [63]

Abbreviations: CRC, colorectal cancer; mCRC, metastatic colorectal cancer, MSS, microsatellite stable; PI3K, phosphatidylinositol 3-kinase; *PIK3CA*, phosphatidylinositol 3-kinase catalytic subunit alpha; *PTEN*, phosphatase and tensin homolog; RAS-WT, rat sarcoma virus—wild type.

**Table 3:** Selected phase I and II trials of isoform-specific PI3K inhibitors for CRC treatment.

Drug	Condition	Phase	Status	NCT, Reference
PI3Kα inhibitors				
Alpelisib (BYL719)	*PIK3CA*-mutated advanced solid tumors	I	Completed	NCT01219699 [67]
	Advanced solid tumors	I	Completed	NCT01449058
	*BRAF*-mutated mCRC	I/II	Completed	NCT01719380 [68]
	*PIK3CA*-mutated mCRC	I/II	Active, not recruiting	NCT04753203 [69]
Inavolisib (GDC-0077)	mCRC	I	Recruiting	NCT04929223
Serabelisib (TAK-117)	Advanced solid tumors	I	Completed	NCT01449370 [70]
	Advanced solid tumors	I/II	Unknown	NCT04073680
	*PIK3CA*-mutated advanced solid tumors	I	Active, not recruiting	NCT05300048
TOS-358	Solid tumors	I	Recruiting	NCT05683418
PI3Kβ inhibitors				
AZD8186	Advanced solid tumors	I	Completed	NCT01884285 [71]
	*PTEN/PIK3CB*-altered advanced solid tumors	I	Active, not recruiting	NCT03218826
GSK2636771	*PTEN*-deficient advanced solid tumors	I/II	Completed	NCT01458067 [72,73]
	Advanced refractory Solid Tumors	I	Active, not recruiting	NCT02465060
SAR260301	Advanced cancers	I	Completed	NCT01673737 [74]
PI3Kδ inhibitors				
Parsaclisib (INCB050465)	Advanced solid tumors	I	Completed	NCT02646748

Abbreviations: BRAF, v-raf murine sarcoma viral oncogene homolog B1; CRC, colorectal cancer; mCRC, metastatic colorectal cancer; *PIK3CA*, phosphatidylinositol 3-kinase catalytic subunit alpha; PIK3CB, phosphatidylinositol 3-kinase catalytic subunit beta; PI3Kα, phosphatidylinositol 3-kinase alpha; PI3Kβ, phosphatidylinositol 3-kinase beta; PI3Kδ, phosphatidylinositol 3-kinase delta; *PTEN*, phosphatase and tensin homolog.

**Table 4:** Selected phase I and II trials of dual PI3K/mTOR inhibitors for CRC treatment.

Drug	Condition	Phase	Status	NCT, Reference
Apitolisib (GDC-0980)	Refractory solid tumors	I	Completed	NCT00854152 [81]
	Advanced solid tumors	I	Completed	NCT01332604
Bimiralisib (PQR309)	Advanced solid tumors	I	Completed	NCT01940133 [82]
Dactolisib (BEZ-235)	Advanced solid tumors	I/II	Completed	NCT00620594 [83]
	Advanced solid tumors	I	Completed	NCT01195376 [84]
	Metastatic or locally advanced solid tumors	I	Completed	NCT01285466 [85]
	Advanced solid tumors	I	Completed	NCT01337765
	Advanced solid tumors	I	Completed	NCT01343498 [86]
	Advanced solid tumors	I	Terminated	NCT01508104 [87]
DS-7423	Advanced solid tumors	I	Completed	NCT01364844 [88]
Gedatolisib (PKI-587)	Solid tumors	I	Completed	NCT00940498 [89]
	Advanced cancer	I	Terminated	NCT01347866 [90]
	mCRC	II	Terminated	NCT01925274
PWT33597 Mesylate	Advanced malignancies	I	Completed	NCT01407380
Samotolisib (LY3023414)	Advanced cancer	I	Completed	NCT01655225 [91]
	Advanced or metastatic solid tumors	I	Completed	NCT02784795 [92]
Voxtalisib (XL-765)	Solid tumors	I	Completed	NCT00485719 [93]
	Solid tumors	I	Completed	NCT00777699 [94]
	Locally advanced or metastatic solid tumors	I	Completed	NCT01390818 [95]

Abbreviations: CRC, colorectal cancer; mCRC, metastatic colorectal cancer; mTOR, mammalian target of rapamycin; PI3K, phosphatidylinositol 3-kinase.

**Table 5:** Selected phase I, II, and III trials of Akt and mTOR inhibitors for CRC treatment.

Drug	Condition	Phase	Status	NCT, Reference
Akt inhibitors				
Afuresertib (GSK2110183)	Solid tumors	I	Completed	NCT01476137 [98]
Enzastaurin (LY317615)	Recurrent CRC	II	Completed	NCT00437268
	mCRC	II	Completed	NCT00612586 [99]
	mCRC	II	Completed	NCT00192114
Ipatasertib (GDC-0068)	Locally advanced or metastatic solid tumors	I	Completed	NCT01562275 [100]
MK-2206	Advanced solid tumors	I	Completed	NCT00670488 [101]
	Advanced CRC	II	Completed	NCT01333475 [102]
	*PTEN* loss and *PIK3CA* mutated mCRC	II	Completed	NCT01802320 [103]
Nelfinavir	Locally advanced CRC	I/II	Completed	NCT00704600
Perifosine (KRX-0401)	mCRC	II	Completed	NCT00398879 [104]
	Refractory advanced CRC	III	Completed	NCT01097018 [105]
mTOR inhibitors				
Everolimus (RAD001)	mCRC	II	Completed	NCT00337545
	Refractory mCRC	II	Completed	NCT00419159 [106]
	mCRC	I	Completed	NCT00478634 [107]
	mCRC	I/II	Completed	NCT00522665 [108]
	Refractory mCRC	II	Completed	NCT00597506 [109]
	CRC	I/II	Completed	NCT01047293 [110]
	mCRC	I/II	Completed	NCT01058655 [111]
	KRAS WT mCRC	I/II	Completed	NCT01139138 [112]
	Refractory mCRC	I	Completed	NCT01154335 [113]
	mCRC	II	Completed	NCT01387880 [114]
	Solid tumors	I	Completed	NCT02890069
	mCRC	II	Recruiting	NCT05725200
Nab-rapamycin (ABI-009)	Advanced carcinoma	I/II	Completed	NCT03190174 [115]
	Advanced or mCRC	I/II	Completed	NCT03439462 [116]
Rapamycin (Sirolimus)	Advanced malignancies	I	Completed	NCT00375245 [117]
	Rectum cancer	I/II	Completed	NCT00409994 [118]
	Advanced malignancies	I	Completed	NCT00707135 [117]
	Solid tumors	I	Completed	NCT01522820
Temsirolimus (CCI-770)	Refractory CRC	I	Completed	NCT00593060
	KRAS mutated mCRC	II	Completed	NCT00827684 [119]
	Advanced cancers	I	Completed	NCT01183663 [120]
	Hepatic metastatic cancer	I	Recruiting	NCT03203525

Akt, protein kinase B; CRC, colorectal cancer; mCRC, metastatic colorectal cancer; mTOR, mammalian target of rapamycin; PI3K, phosphatidylinositol 3-kinase; *PI3KCA*, phosphatidylinositol 3-kinase catalytic subunit alpha; *PTEN*, phosphatase and tensin homolog; RAS-WT; kirsten rat sarcoma virus—wild type.

## Data Availability

Data sharing is not applicable to this article as no new data were created or analyzed in this study.
